# The Sensory Ecology of Tsetse Flies: Neuroscience Perspectives on a Disease Vector

**DOI:** 10.1111/ejn.70377

**Published:** 2026-01-28

**Authors:** Andrea Adden, Lucia L. Prieto‐Godino

**Affiliations:** ^1^ The Francis Crick Institute London UK

**Keywords:** *Diptera*, *Drosophila*, evolution, *Glossina*, hematophagy, viviparity

## Abstract

Tsetse flies (*Glossina* sp.) are important disease vectors with unique biology that makes them fascinating models to study the evolution of behaviour and its underlying neural circuits. They evolved blood‐feeding in an independent event from mosquitoes, and unlike most insects, give birth to a single live offspring—rather than laying eggs. Given their impact on public health, they have been extensively studied with a strong focus on vector control. However, information on their sensory ecology and neurobiology is thinly spread across the literature. Here, we review over a hundred years of literature on tsetse sensory systems, including olfaction, vision, audition, taste, thermosensation and mechanosensation, in the context of the behaviours they drive, including host‐finding, blood‐feeding and mating. We embed the available data within our more detailed understanding of the sensory systems of the vinegar fly *Drosophila melanogaster* and other diptera. This sets the stage for future work on how tsetse find their hosts and reproduce, opening new avenues to understand how their sensory systems function and evolve, which in turn will inform better control strategies to reduce the burden of the diseases they transmit.

## Graphical abstract

Tsetse flies (*Glossina* sp.) are important disease vectors that feed on vertebrate blood. Host‐seeking depends on a combination of sensory systems, from long‐range senses like olfaction and vision, to shorter‐range senses such as audition, mechanosensation, thermosensation and taste. We embed over 100 years of tsetse research in our more detailed understanding of the sensory systems of *Drosophila melanogaster* and other diptera, to explore how tsetse behaviours evolved and function.

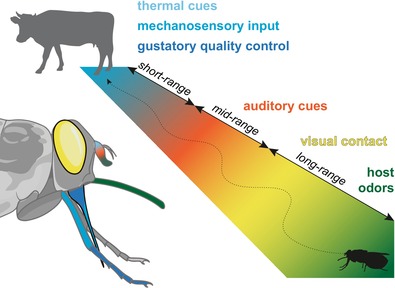

**Abbreviations**
ATP: adenosine triphosphate
CvS: cervical sclerites
Cx: coxa
F: funicle (third antennal segment)
GR: gustatory receptor
IR: ionotropic receptor
JO: Johnston's organ
La: lamina (neuropil of the optic lobe)
LC: labrocibarial receptor
Lo: lobula (neuropil of the optic lobe)
LoP: lobula plate (neuropil of the optic lobe)
Me: medulla (neuropil of the optic lobe)
OBP: odorant‐binding protein
OR: odorant receptor
Orco: odorant receptor coreceptor
OSN: olfactory sensory neuron
P: pedicel (second antennal segment)
Pb: probasisternum
PM: prosternal membrane
Pr: presternum
R1‐8 p/y: rhabdomere 1‐8 pale/yellow
Rh: rhodopsin
Sa: sacculus
Sc: scapes (first antennal segment)
SP: sensory pit
TRP: transient receptor potential channel
UV: ultraviolet

## 1 Introduction

Tsetse flies (genus: *Glossina*) are exclusively blood‐feeding flies widely distributed across sub‐Saharan Africa. They are vectors for trypanosomes (*Trypanosoma* sp.), which cause sleeping sickness among humans and the equivalent disease, nagana, among cattle (Kuzoe and Schofield 2004). While sleeping sickness is treatable, it often results in lifelong disability. Nagana is often fatal. Consequently, tsetse flies have an enormous impact on public health and economic development in sub‐Saharan Africa (Alsan 2015). However, surprisingly little is known about the sensory basis of tsetse behaviours. This review aims at consolidating what we know about their sensory ecology and highlighting open questions for the future, as well as their potential applications for vector control.

**FIGURE 1:**
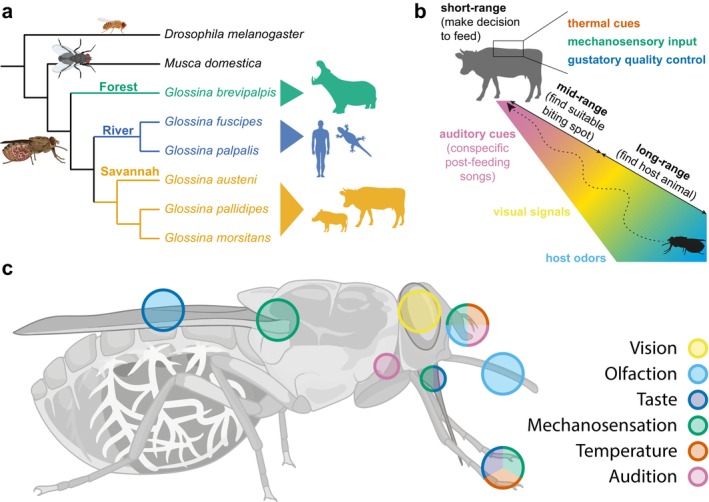
Overview over Glossina sp. (a) The phylogenetic tree of *Glossina*. Tsetse can be subdivided into three subgroups: the forest (‘fusca’), riverine (‘palpalis’) and savannah (‘morsitans’) groups. (b) The host‐seeking sequence as described for *Glossina*. (c) Distribution of sensory systems.

Unlike in several mosquitoes, where only the female feeds on blood before oviposition, both male and female tsetse flies feed exclusively on vertebrate blood. Different species inhabit different habitats and specialise in different hosts. They can be subdivided into three broad phylogenetic groups with divergent ecologies (Figure 1a): The savannah species group (‘Morsitans’ group, including *G. morsitans*, *G. austeni* and *G. pallidipes* ) inhabit grassland and open woodland, feed predominantly on domestic and wild cattle and pigs (such as warthogs) and are the main transmitters of nagana. The riverine group (‘Palpalis’ group, including *G. palpalis* , and *G. fuscipes* ) live along rivers and lake shores, preferentially feeding on humans and reptiles, and are the main vectors of human trypanosomiasis (sleeping sickness). Finally, the forest group (‘Fusca’ group, including *G. brevipalpis*), feed on larger mammals, such as hippopotamus, and are of less epidemiological importance (Kuzoe and Schofield 2004). Field trapping experiments showed that in the wild these species groups also differ in their responsiveness to different sensory stimuli. For example, while all species are attracted to visual targets in the field, adding host odours to the traps substantially increased the trapping success for savannah group species, but less so for riverine species (Green 1994; Torr and Vale 2015). However, a confounding factor in those experiments may have been the nature of the host odours, as only ox odours were tested, whereas other field studies found that different species have divergent preferences for the odours of different hosts (Mohamed‐Ahmed 1998; Omolo et al. 2009). A computational model attempted to explain the divergent preference of different species towards olfactory and visual targets based on differences in the vegetation density of the habitats. Specifically, the model found that the dense vegetation of riverine environments restricts fly movement, diminishing the effectiveness of odour baits (Vale et al. 2014). Additionally, as we discuss below, it also seems that different tsetse species have divergent visual preferences with respect to both the targets' shape and colour (Rayaisse et al. 2011; Tirados et al. 2011). Therefore, it is likely that different tsetse species have evolved their sensory systems in adaptation to the habitats they occupy, both in terms of the relative importance of different sensory modalities (e.g., vision vs. olfaction) and also in terms of stimulus preference and specificity.

The sensory ecology of tsetse might also differ from that of other blood‐feeding insects, such as mosquitoes, given their divergent reproductive strategy (reviewed in (Benoit et al. 2015)). Like all Hippoboscoid flies, tsetse are viviparous. The female gets pregnant with one larva at a time, which develops from first to third instar inside the female’s uterus as it feeds on ‘milk’ secreted by the mother from modified accessory glands (Denlinger and Ma 1974). Females give birth to a single third instar larva that immediately burrows into the ground to pupate. The adult hatches 30 days later (Benoit et al. 2015). Female tsetse only mate once, store the received sperm in their spermathecae and give birth to approximately eight to 10 larvae during their lifetime (Benoit et al. 2015). Therefore, tsetse flies invest in the quality, rather than the quantity, of their offspring. This has two important consequences for tsetse sensory biology. First, it is possible that females invest significant sensory resources to choose an adequate partner, as well as a suitable larviposition site. Second, host‐finding and feeding strategies are likely to have evolved to maximise the female’s survival. For example, it has been speculated that savannah and riverine species differ in their attraction to humans due to their different ‘risk‐taking’ behaviours: Humans are considered high risk for tsetse, given our skills at swatting them, but riverine species are more willing to take risks, perhaps due to the low density of hosts in the habitats they inhabit (Torr and Vale 2015).

Host‐seeking behaviour in tsetse flies follows a stereotyped sequence (Figure 1b–c), beginning with long‐range olfactory cues that guide the fly towards hosts, followed by visual cues that help locate the target (Vale 1974a; Torr and Vale 2015). Upon entering a closer range around the host animal, the fly can then use mid‐range auditory cues to identify a suitable biting site (Popham et al. 1976). Finally, short‐range cues—a combination of thermal, mechanical and gustatory stimuli—help the fly choose the exact biting location and induce the fly to bite and feed. A similar, but less well‐described sequence of events precedes mating, with an initial visual contact inducing the male to chase a female (Brady 1991). More recently, volatile semiochemicals emitted by females were found to attract males (Ebrahim et al. 2023). Although there is still a gap in our knowledge when it comes to courtship, we know that copulation initiation depends on a gustatory cue—a contact pheromone—and is also controlled by mechanical and/or auditory stimuli (Wall and Langley 1993). Even less information is available when it comes to larviposition behaviours, but soil humidity, temperature and chemical profile all appear to be important cues for the female when choosing a site to lay its larva (Muzari and Hargrove 2005; Renda et al. 2016; Adden et al. 2023). Understanding the interplay between cues in these multisensory behaviours and the underlying neural mechanisms in more detail will open up new possibilities for disrupting tsetse behaviour, enlarging our toolkit for vector control. The availability of a genome for six tsetse fly species (Figure 1a; (Attardo et al. 2019)) provides a timely opportunity to delve deeper into the neural and molecular basis of tsetse behaviours and their evolution. Here, we review what is known about tsetse sensory ecology, highlight gaps in our current knowledge about these important disease vectors and suggest possible avenues for future research.

## 2 Senses for Long‐Range Cues: Olfaction

Hosts constantly emit odour volatiles through breathing or perspiration. In addition, host‐associated excretions, such as urine, contain volatile compounds. The combination of these volatiles, termed kairomones, is specific to each host species and can therefore serve to identify the host. Odours disperse in characteristic plumes, which can stretch across several hundred metres, depending on environmental conditions (Wall and Perry 1987; Murlis et al. 2000). Thus, a tsetse fly intercepting an odour plume can use it to find the animal at its source (Gibson and Young 1991), a behaviour that has been exploited for vector control to enhance tsetse fly traps. For example, one study found that adding odour lures to visual traps increased the trapping rate 20‐fold in the savannah species *G. morsitans* and *G. pallidipes* (Vale 1974a). Here, we first describe what is known on tsetse olfactory ecology and then delve into the neural bases underlying odour detection in tsetse.

Blood meal analysis of captured flies has identified the major hosts of each tsetse species. For example, savannah species were found to feed mostly on Suidae (pigs and relatives), with some variations by species, location and likely host availability. *G. austeni* seems to prefer bushpig, while *G. morsitans* prefers warthog and ruminants, and *G. pallidipes* feeds mostly on ruminants. In contrast, *G. brevipalpis* uses hippopotamus as main hosts, and the palpalis group was found to be more of a generalist with a significant proportion of blood meals from primates and reptiles, in addition to pigs and ruminants (Weitz 1963; Clausen et al. 1998; Muturi et al. 2011). Beyond this, trapping experiments found that *G. fuscipes* is particularly attracted to lizards (Mohamed‐Ahmed 1998; Omolo et al. 2009), while *G. morsitans* has a preference for buffalo and ox and is deterred from waterbuck (Gikonyo et al. 2002; Wachira et al. 2020) (Figure 1a). Ingenious early experiments determined that odour was a main attractant to host animals while also being a potent repellent from nonhosts: Vale and Hargrove (1979) placed electric nets around a vent releasing air from an underground pit containing host animals. The experimenters were watching from a separate pit, from which odour was pumped and released 50 m away, to avoid any repellent effect human odour might have on tsetse (Vale 1974b; Hargrove 1976). Using this setup, it was demonstrated that cattle odour has a differential attractiveness to different tsetse species (Vale and Hargrove 1979).

Since then, kairomones that are responsible for host attraction across species have been identified as 1‐octen‐3‐ol (Hall et al. 1984), acetone (Vale and Hall 1985) and a variety of phenolic compounds (Hassanali et al. 1986; Bursell et al. 1988; Vale et al. 1988), among others. Follow up experiments also identified host odours that repel tsetse. Among these, the ‘waterbuck repellent blend’ is widely used, which consists of a specific ratio of pentanoic acid, guaiacol, d‐octalactone and geranylacetone. It was shown to be most effective against *G. morsitans*, while *G. pallidipes* and *G. fuscipes* are repelled to a lesser extent (Gikonyo et al. 2002; Mbewe et al. 2019). This highlights differences in olfactory preferences across species. Subsequent efforts have focused on identifying the neural substrates of repellent detection (Gikonyo et al. 2002; Diallo et al. 2020; Wachira et al. 2020) and finding simpler and more potent repellent compounds (Mbewe et al. 2019). Simultaneously, there is an ongoing search for potent attractive components to use in push–pull strategies that use repellents to deter flies from livestock or humans, while using attractants to lead the flies to traps (Madubunyi et al. 1996; Omolo et al. 2009; Rayaisse et al. 2010; Terfa et al. 2014). These studies focus on expanding our knowledge of tsetse chemical ecology to exploit species‐specific host odour preferences for vector control.

**FIGURE 2:**
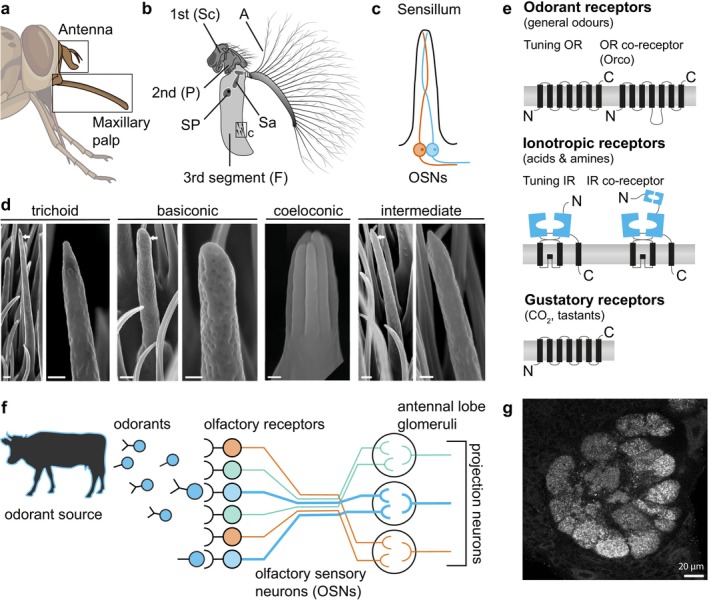
The olfactory system. (a) Olfactory receptors sensitive to volatile compounds are found on the antenna and the maxillary palps. (b) The antenna consists of three segments: the scape (1st, Sc), pedicel (2nd, P) and funicle (3rd, F) with the arista (A). Two invaginations in the funicle are termed the sacculus (Sa) and sensory pit (SP). Drawn after (Jobling 1933). (c) The antennal surface is covered in sensilla, which are innervated by the dendrites of up to four olfactory sensory neurons (OSNs). (d) Olfactory receptors expressed in the OSNs belong to three different groups: Odorant receptors (ORs) sense various volatile odours, ionotropic receptors (IRs) sense acids and amines, and gustatory receptors (GRs) sense CO_2_ as well as contact chemicals. (e) Sensilla types identified on the surface of the 3rd antennal segment in *G. m. morsitans*. Reproduced from (Chahda et al. 2019). (f) Odorants from host animals and other sources bind to olfactory receptors, activating OSNs. OSNs expressing the same receptor type project to the same glomerulus in the antennal lobe, the primary olfactory neuropil in the brain. Projection neurons integrate information from an entire glomerulus and project to higher processing centres. (g) Confocal image of a tsetse antennal lobe, stained with an anti‐synapsin antibody. Individual glomeruli are easily discernible. Scale bar = 20 μm.

Tsetse display other odour‐guided behaviours beyond host seeking. Mating has been shown to depend on multiple cues, including vision, taste and olfaction. Recent work identified three volatile sex attractants in *G. morsitans*: methyl palmitate, methyl oleate and methyl palmitoleate. These compounds are found in females, are attractive to male flies and elicit electrophysiological responses from olfactory sensory neurons (OSNs) in both sexes, albeit with more sensitivity in males for some of the volatiles. Interestingly, the compounds seem to be species‐specific, as they did not elicit significant behavioural or electrophysiological responses in *G. fuscipes* (Ebrahim et al. 2023). Sex pheromones, combined with insecticides or traps, have potent vector control potential in the field, as demonstrated by recent advances in vector control of the sandfly *Lutzomyia longipalpis* (Hamilton 2022). Another behaviour that has been suggested to be guided by olfactory cues is the female larviposition site choice (Saini et al. 1996; Gimonneau et al. 2021, 2024). Larvae excrete species‐specific compounds during pupation: pentadecane for *G. morsitans morsitans* and dodecane for *G. morsitans centralis* (Saini et al. 1996). Females of each species can detect its corresponding volatile through OSNs in the antennae (Figure 1c and Figure 2a) as revealed by electroantennogram recordings, but the identity of the sensillum and receptors remains unknown (Leonard and Saini 1993; Saini et al. 1996). Importantly, while pupae of the same species are often found clustered in the field (Renda et al. 2016), the significance of larval pheromones in attracting females to suitable larviposition sites in the field remains unclear (Hargrove et al. 2021), and a recent study concluded that pheromones are unlikely to play a role in female larviposition site choice under naturalistic conditions (Adden et al. 2023).

The main olfactory organs of tsetse and most insects are the third antennal segment and the maxillary palps (Figure 1c and Figure 2a–b). Structurally, both organs resemble the well‐described homologous organs in the vinegar fly *Drosophila melanogaster* (Figure 2a–b; (Vosshall 2000; Chahda et al. 2019)). OSNs expressing olfactory receptors are organised inside cuticular structures called sensilla (Figure 2c; (Schmidt and Benton 2020)). In *Drosophila*, each sensillum contains between one and four OSNs arranged in a stereotyped manner (Vosshall 2000). Four types of morphologically distinct sensilla have been identified in the tsetse antennae by homology to its *Drosophila* counterparts: trichoid, basiconic, coeloconic and intermediate (Figure 2d; (Isaac et al. 2015; Chahda et al. 2019)). In *D. melanogaster* , these broad anatomical types are further subdivided by the stereotypic combination of olfactory receptors expressed in the OSNs within each sensillum (Couto et al. 2005; Silbering et al. 2011). Similarly, in tsetse, three types of trichoid and four types of basiconic sensilla have been identified based on differences in odour tuning (Soni et al. 2019). However, it is likely that there are many more sensillum types per class, as this study only examined two regions of the tsetse antennal surface.

The odour sensitivity of OSNs is conferred by the expression of olfactory receptors. Three main families of olfactory receptors are expressed in the insect antenna: Odorant Receptors (ORs), Ionotropic Receptors (IRs) and Gustatory Receptors (GRs) (Figure 2e); (Wicher and Miazzi 2021). Despite their name, some GRs are expressed in olfactory organs, where they form a complex that detects CO_2_. OR complexes are heterotetramers of tuning ORs and the co‐receptor Orco (Butterwick et al. 2018; del Mármol et al. 2021). Receptors of the IR family are heterotetramers of ligand‐sensitive IR subunits and one or several co‐receptors, which differ depending on the receptor's sensitivity: The co‐receptor of acid‐sensing IRs is IR8a, while the co‐receptor of amine‐sensing IRs is IR25a (Benton et al. 2009; Rytz et al. 2013). However, it is worth noting that there are members of the IR family that function in other sensory modalities beyond olfaction (Senthilan et al. 2012; Enjin et al. 2016; Budelli et al. 2019). All three olfactory receptor families have been identified in the genome of the six sequenced tsetse fly species, displaying interesting evolutionary patters of gene gain and loss, as well as signatures of selection across tsetse species (Obiero et al. 2014; Macharia et al. 2016; Kabaka et al. 2020). This supports behavioural evidence that different tsetse species have evolved their olfactory system in adaptation to their different ecological niches. Additionally, experimental work has confirmed the expression of some of these olfactory receptors in the antenna of *G. morsitans* and, for a small subset of receptors, identified several ligands that activate them, which include host odours (Chahda et al. 2019).

When odours enter an olfactory sensillum through its pores, they dissolve in the sensillar lymph, which contains odorant‐binding proteins (OBPs) alongside other proteins. These OBPs are thought to perform a number of functions, from odour transport in the sensilla to odour buffering (Larter et al. 2016). OBPs have been identified in the genome of all six tsetse fly species, and the expression of a subset was confirmed in the antennae and legs of *G. morsitans* and *G. fuscipes* (Liu et al. 2010; Kabaka et al. 2020). Furthermore, in *G. fuscipes* gene silencing via RNAi feeding demonstrated that an OBP predicted to interact with the host odour 1‐octen‐3‐ol is required for the attraction of this tsetse species towards the odour (Diallo et al. 2021). The OBP expression pattern in tsetse resembles that found in *D. melanogaster* , confirming that the three main sensilla types, basiconic, coeloconic and trichoid, are similar between *D. melanogaster* and tsetse both in morphology and molecular identity. For example, the homologue of *Drosophila*'s pheromone‐binding OBP *Lush* is expressed—as in *D. melanogaster* —at the base of trichoid sensilla in tsetse (Chahda et al. 2019). In fruit flies, trichoid sensilla house OSNs that sense volatile pheromones, and recent work suggests that they fulfil a similar role in tsetse. Some tsetse trichoid sensilla were found to respond to tsetse sex attractants (Ebrahim et al. 2023) as well as to other compounds known to act as pheromones in other insects, such as the *D. melanogaster* sex pheromone methyl laurate (Dweck et al. 2015) and the giant hornet alarm pheromone 2‐pentanol (Ono et al. 2003; Soni et al. 2019). Interestingly, the OSNs housed in tsetse trichoid sensilla also respond to odours not thought to act as pheromones, including host odours (Soni et al. 2019; Ebrahim et al. 2023).

The tsetse antenna contains two invaginations, termed sensory pit and sacculus. The sensory pit is located medially on the antenna and contains basiconic type II sensilla. In *G. morsitans* these sensilla contain two OSNs, each OSN expressing either GmmOr6 or GmmOr9. GmmOr9 is sensitive to several tsetse attractants classically used in odour‐baited traps (Chahda et al. 2019), suggesting that the sensory pit, which is absent in *Drosophila*, plays a role in host‐finding behaviours. The sacculus opens to the lateral side of the antenna and consists of a dorsal and a ventral chamber. The dorsal chamber contains basiconic type II sensilla and the ventral chamber contains coeloconic sensilla, which can be grooved or smooth. For comparison, the *D. melanogaster* sacculus has three chambers, with the first two containing smooth coeloconic sensilla. These sensilla are innervated by neurons expressing members of the IR family and are sensitive to humidity (Enjin et al. 2016). The third chamber contains grooved coeloconic sensilla, which host OSNs that express other members of the IR family, making them sensitive to acids (Ai et al. 2010). In the tsetse antenna, the expression of IRs has not been investigated to date. However, two OBPs known from *D. melanogaster* to be coeloconic‐specific are also associated with coeloconic sensilla in tsetse, indicating that the function of coeloconic sensilla in the sacculus may be conserved (Chahda et al. 2019).

The characterisation of the tsetse antenna's organisation at the molecular level is still in its infancy, with recent work analysing the expression pattern and responses of a subset of receptors (Chahda et al. 2019; Soni et al. 2019). Studies performing electrophysiological recordings from the antennae of *G. morsitans*, *G. pallidipes* and *G. fuscipes* found that a large proportion of sensilla on the antennal surface respond to 1‐octen‐3‐ol, a host emanate (Voskamp, Everaarts, and Den Otter 1999; Voskamp, van der Goes van Naters, and Den Otter 1999; Soni et al. 2019), suggesting a key conserved ecological role for this odour. Although the molecular bases for this sensitivity remain enigmatic, it is possible that a large proportion of sensilla share an OSN expressing the same receptor, similar to what has been observed in *D. melanogaster* coeloconic sensilla: Here, three out of the five classes of coeloconic sensilla contain an OSN expressing the receptor DmIR75d, while the other two classes both contain an OSN expressing receptor DmOR35a (Silbering et al. 2011; Prieto‐Godino et al. 2017). Another interesting feature of the tsetse olfactory system is that its olfactory receptor repertoire is smaller than the one found in *Drosophila* species (Macharia et al. 2016). This is largely due to a reduced number of ORs across *Glossina* sp., reported to be between 37 and 42 (depending on the tsetse species) compared to 60 in *D. melanogaster* . In contrast, the olfactory IR repertoire is better conserved. Most tsetse species have at least one copy of each of the 15 olfactory tuning IRs described in *D. melanogaster* , with a few lineage‐specific copy number variations. One exception is IR75a, IR75b and IR75c, which are Drosophilid‐specific duplications (Prieto‐Godino et al. 2017): In tsetse, all species have two copies of an independent lineage‐specific duplication of IR75a (Kabaka et al. 2020; Prieto‐Godino et al. 2017). The number of GR subunits forming the CO_2_ receptor is two in Drosophilids, three in mosquitoes and four in tsetse. In mosquitoes, it was shown that the third GR subunit of the CO_2_ receptor—missing in *D. melanogaster* —modulates the response of this receptor complex (Kumar et al. 2020). The function and expression of these subunits in tsetse remain enigmatic. In mosquitoes, both CO_2_ detection by the GR complex and detection of acids by IR receptors have been shown to be critical for vertebrate host finding (Raji et al. 2019; Ray et al. 2023). This might explain the lower investment of tsetse in general purpose OR receptors, while maintaining and expanding its IR and GR repertoire.

In vertebrates and *Drosophila*, as a rule, each OSN expresses a single functional olfactory receptor type. Furthermore, all OSNs expressing the same receptor type project to the same glomerulus in the antennal lobe (AL)—the brain's primary olfactory centre (Figure 2f–g). This organisation leads to a good correlation between the number of expressed olfactory receptors and the number of AL glomeruli (Couto et al. 2005; Fishilevich and Vosshall 2005). Interestingly, this rule does not seem to hold for mosquitoes, where a recent study found the number of expressed receptors to be at least twice the number of glomeruli (Herre et al. 2022). This study also demonstrated coexpression of multiple functional receptor types, as well as convergence onto individual glomeruli from OSNs expressing different co‐receptor subunits. In *D. melanogaster* , co‐expression of co‐receptors is also common, but co‐expression of functional receptor complexes belonging to the IR and OR families is rarer (Task et al. 2022; Benton et al. 2025). In tsetse the exact relationship between glomeruli and olfactory receptors remains unknown, but initial data suggest an approximate one‐to‐one correspondence as seen in *D. melanogaster* (Soni et al. 2019).

It has long been recognised that host‐finding behaviour involves both vision and olfaction, and that the two senses strongly interact: When following an odour, tsetse flies are attracted towards visual targets (Vale 1974a; Torr 1989) and keep searching in the odour plume until a visual target is found (Vale 1974a; Gurba et al. 2012). A similar behaviour has been described in mosquitoes, where olfactory CO_2_ detection elicits attraction towards visual targets (Van Breugel et al. 2015; Vinauger et al. 2019). Further, field observations revealed that only half of the flies approaching an odour‐baited trap ended up being trapped, indicating that odour alone is not always sufficient for inducing landing behaviour (Rayaisse et al. 2010). These findings emphasise that, to fully understand the long‐range component of host‐seeking behaviours in tsetse, we must understand both their olfactory and visual ecology.

## 3 Senses for Long‐Range Cues: Vision

**FIGURE 3:**
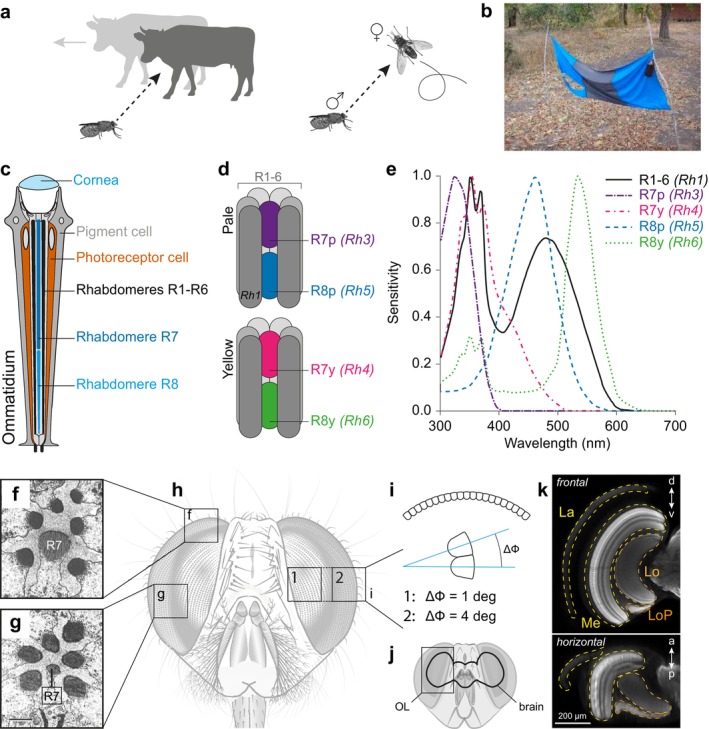
The visual system. (a) Visual behaviours of tsetse include host‐seeking and mate detection. (b) Tsetse are strongly attracted to blue traps. (c) An ommatidium of the fly compound eye. Light passes through the lens and travels through the light guide, where it is absorbed by the rhodopsins expressed in photoreceptor rhabdomeres. The central stacked rhabdomeres R7 and R8 are surrounded by rhabdomeres R1‐6. (d) Ommatidia are subdivided into *yellow* and *pale* ommatidia based on their rhodopsin expression pattern. Yellow ommatidia express Rh3 (R7) and Rh5 (R8), whereas pale ommatidia express Rh4 (R7) and Rh6 (R8). R1‐6 express Rh1. (e) Absorption spectra of fly photoreceptors (Santer 2015). (f,g) R7 and R8 are enlarged in the dorsal rim of the *Glossina* eye as compared to the remainder of the eye. Images reproduced from (Hardie et al. 1989). (h,i) The interommatidial angle is lower in the frontally‐located acute zone (1) than in the periphery (2). (j) The optic lobes (OL) are the primary visual neuropils, and make up more than 60% of the brain. (k) Confocal image of a tsetse optic lobe, stained with an anti‐synapsin antibody. The OL consists of four neuropils: the lamina (La), medulla (Me), lobula (Lo) and lobula plate (LoP).

The sense that has received the most attention in tsetse research during the past century is vision. Until the importance of odours in host attraction was acknowledged, trapping efforts employed visual targets impregnated with glue or insecticide. Being day‐active (Brady 1972), many tsetse behaviours have crucial visual components, including host‐seeking and mate detection, for which the flies rely on sensing the colour, movement and/or shape of a visual target (Figure 3a–b). For example, at a spatial resolution of 1° (frontal eye, Figure 3i), a tsetse would be able to distinguish a 2‐m tall human from a distance of approximately 115 m—a useful visual aid when navigating towards the source of an odour plume.

Tsetse fly eyes follow the typical dipteran organisation of neural superposition compound eyes (Hardie et al. 1989). Behind the lens of each facet (rhabdom), six photoreceptor cells (R1 to R6) are arranged concentrically around two central, stacked photoreceptors (R7 and R8). The light‐sensitive structures of photoreceptor cells are called rhabdomeres—brushes of microvilli in which photosensitive opsins are expressed. As a rule, R7 and R8 are smaller than the surrounding rhabdomeres, except in the dorsal‐most rhabdoms, in which the two central rhabdomeres are enlarged (Figure 3c–d; (Hardie et al. 1989)). The optical axes of the eight photoreceptor cells are not aligned with each other; rather, they are aligned with photoreceptor cells in the neighbouring rhabdoms. If the information relayed by the photoreceptor cells within one facet was to be integrated, this arrangement would lead to a blurred pixel. However, in a neural superposition eye, the axons from rhabdomeres located in neighbouring facets that view the same point in space are coupled via gap junctions and project onto the same lamina cartridge. Signals from the same point in space are therefore integrated in a so‐called neuro‐ommatidium, allowing the fly to retain high spatial resolution while maintaining high light sensitivity (Kirschfeld 1967; Lunau 2014).

The molecular basis of fly vision has been examined in detail in several species (Schmitt et al. 2005; Montell 2012). Across species, R1‐6 express the visual pigment Rhodopsin 1 (Rh1), which peaks in the green and ultraviolet (UV). These photoreceptor cells feed into motion vision pathways. Flies have two distinct classes of R7/8, termed ‘yellow’ and ‘pale’, which form the basis for colour vision (reviewed in Lunau 2014). Yellow R7/8 express Rh4 (long UV) and Rh6 (green), while pale R7/8 express Rh3 (short UV) and Rh5 (blue), respectively (Figure 3d–e). Local circuit comparison across R7/8 gives rise to spectral opponency at the synaptic output of these photoreceptors (Heath et al. 2020) that, like in vertebrates (Yoshimatsu et al. 2021), could be the basis for spectral feature channels in flies. Early electrophysiology studies showed that the spectral sensitivities of tsetse fly photoreceptors resemble those of other flies (Hardie et al. 1989). A comparative genomic analysis showed that, like other Calyptratae, Glossinids lack Rh4 (long UV), while Rh5 was shown to have undergone diversifying selection within Glossinids, which may provide a basis for diverging colour preferences (Attardo et al. 2019; Olafson et al. 2021). Behaviourally, the savannah species *G. m. morsitans* has been shown to be attracted to monochromatic UV, blue and red light in the lab (Green and Cosens 1983), while in the field species of the savannah and riverine subgroups are strongly attracted to black and blue, but not UV or green reflecting surfaces (Green and Flint 1986; Lindh et al. 2012; Santer 2014). Again, this is in line with colour preferences of other biting flies (Santer et al. 2023).

The general preference for blue has been interpreted in different ways, with some evidence to support the hypothesis that a host‐sized blue target may resemble a host animal most closely when compared to surrounding vegetation (Santer 2015; Santer et al. 2023). It was also suggested that the fly may interpret blue as shadows, in which many tsetse species seek shelter during the day (Steverding and Troscianko 2004). Understanding colour vision in tsetse and employing photoreceptor‐based models has already led to improved trap designs by using cloths whose reflectance spectrum matches the spectral sensitivities of tsetse photoreceptors (Santer et al. 2019). This illustrates the importance of a complete understanding of tsetse sensory systems for vector control.

While blue is clearly attractive to tsetse, the discrepancy between laboratory and field studies when it comes to UV has not yet been resolved. In the field, the response of tsetse flies to different colours was assessed by evaluating the trapping efficiency of differently coloured traps (Green and Flint 1986). It is worth noting that the number of flies caught in a trap does not necessarily reflect the true number of flies attracted to the trap, as tsetse are known to circle potential landing sites before alighting, and a significant proportion of flies never lands on the target or enters the trap (Lindh et al. 2009). This behaviour is likely to skew assessments of colour attractiveness. Keeping this in mind, UV‐reflecting cloths were comparatively inefficient at eliciting landing responses ((Green and Flint 1986) for *G. pallidipes* and *G. m. morsitans*, but see (Green 1990) for *G. tachinoides*), despite the flies' strong attraction to monochromatic UV in laboratory experiments (Green and Cosens 1983). However, the fat content of female *G. tachinoides* that landed on UV‐reflecting white cloths was higher compared to those that landed on blue targets that had low UV reflectance (Green 1990). One interpretation of this observation was that fed flies attempt to disperse towards the sky, possibly explaining their increased attraction towards UV (Santer 2015).

One aspect of light that has so far been entirely ignored in field studies is polarisation: Surfaces reflect light with a degree of polarisation that depends partly on the structure of the surface, as well as the angle between the light source, surface and observer (Foster et al. 2018). Previous field studies used different materials, such as cotton and polyester, of different colours, and while their spectral reflectivity was measured, the degree and angle of polarisation of the reflected light was not (Green and Flint 1986; Lindh et al. 2012). However, ignoring polarisation may confound our interpretation of how tsetse responds to different colours. It is now well known that many animals use environmental polarisation cues to detect contrasts, enhancing object detection (e.g., (How et al. 2015; Tedore and Nilsson 2019)), for orientation and navigation purposes (reviewed in (Wehner 2001)), and for communication (Marshall et al. 2019). Within diptera, there is good evidence that tabanid flies and stable flies are attracted to polarised light (Horváth et al. 2008; Blake et al. 2023), and that the coats of animals such as horses and zebras have depolarising properties that can counteract this attraction (Horváth et al. 2010; Egri et al. 2012). As tabanids are closely related to tsetse and are similar in their blood‐feeding lifestyle, it is possible that tsetse use polarisation vision in a similar way. Mosquitoes are also weakly attracted to polarised light, but the sources of polarisation in this context are the water surfaces on which female mosquitoes oviposit (Bernáth et al. 2012). Although polarisation vision has never been systematically assessed in tsetse, it has been postulated that tsetse detect skylight polarisation with their enlarged R7/8 photoreceptors at the dorsal rim of the eye (Figure 3f) (Hardie et al. 1989). This area is reminiscent of the dorsal rim areas found in other insects, in which the photoreceptors are arranged optimally for detecting polarised skylight (Labhart and Meyer 1999). Many insects use skylight polarisation for orientation and navigation (e.g., Heinze and Homberg 2007; el Jundi et al. 2014; Warren et al. 2019), but whether tsetse flies can use this cue for the same purpose remains unknown. Beyond the dorsal rim area, ventral photoreceptors have been demonstrated to have weak to moderate polarisation sensitivity in several *Diptera*, including *Glossina* (Hardie et al. 1989). Understanding the role of polarisation vision in tsetse behaviour may allow us not only to solve the puzzling discrepancy in published data but also to design more efficient trapping systems.

The tsetse eye is flattened frontally, and the photoreceptors located in this area consequently have very small interommatidial angles, giving rise to a characteristic acute zone that offers increased resolution (Figure 3h–i) (Turner and Invest 1973; Gibson and Young 1991). Both males and females have an acute zone, but the binocular overlap in this area is larger in males. Analogous to other *Diptera*, it has been suggested that the acute zone is an adaptation to fast flight, as it allows the fly to obtain maximum information from the frontal optic flow field. During forward flight, the origin of translational optic flow is located frontally, and this area is therefore the least blurred part of the entire field of view. High spatial resolution in this area allows the fly to perform complex tasks during flight, for example, avoiding obstacles, detecting small objects and landing (Borst et al. 2010). Matching these requirements, measurements of the flicker‐fusion frequency confirmed that the eye provides high temporal resolution, making it optimally adapted to analyse optic flow during flight (Turner and Invest 1973; Mebourou et al. 2018). Additionally, it is likely that male tsetse use their acute zone for identifying and chasing potential mates (Gibson and Young 1991). Males take off from their perching site to chase females (Brady 1991), and before intercepting, they follow the female and match her speed. As tsetse flies have a significantly faster forward speed than most other flies, this manoeuvre has been suggested to serve as a diagnostic tool for identifying the female's species (Brady 1991), thus providing a possible mechanism for preventing interspecies mating.

Tsetse flies are attracted to visual targets of specific shapes. In particular, there is a clear difference between oxen‐shaped targets (horizontal rectangles) and human‐shaped targets (upright rectangles). The only tsetse species that is visually attracted to upright targets is *G.* *palpalis palpalis*, a riverine species that feeds on humans (Tirados et al. 2011). All other species tested to date, including the riverine tsetse *G. fuscipes quanzensis*, prefer round, square or horizontal targets, with horizontal targets eliciting the highest landing response (Vale 1974a; Rayaisse et al. 2011; Tirados et al. 2011). Whether this is a learnt preference or hard‐wired in the brain remains unexplored. It is interesting to note that riverine species are attracted to substantially smaller targets than other tsetse flies, matching their ecological preference for lizards (Lindh et al. 2009; Esterhuizen et al. 2011). Furthermore, female tsetse in particular appear to actively avoid landing on targets that are human‐like, possibly because the more dexterous humans present a higher threat to the flies' survival (Vale 1974a).

All visual input is processed in the optic lobes (OL), the primary visual neuropils of the insect brain (Figure 3j), which are subdivided into the lamina, medulla, lobula and lobula plate (Figure 3k). We know from other flies that neurons in these neuropils extract features such as shape, colour, visual motion and polarisation from the environment (Borst et al. 2010). While not yet explored in tsetse, the anatomical organisation of the OL neuropils is well conserved (Figure 3k), suggesting conserved functions.

To conclude, vision and olfaction are the main long‐distance cues that guide a tsetse fly to its host. Once the fly has found a potential host animal, it must then find a suitable feeding site. This task is nontrivial, as not all areas on the host are equally accessible: Dense fur may prevent the stylus from penetrating the skin, whereas skin ripples, flicking ears and swishing tails may dislodge or even kill the fly. The feeding site must therefore be chosen with care, and it has been shown that songs produced by conspecifics may aid in this endeavour. In the following, we will discuss the current state of knowledge on the tsetse auditory system and the behaviours it mediates.

## 4 Senses for mid‐Range Cues: Audition

Tsetse produce sounds during various behaviours, including feeding, mating, larviposition and flight. These sounds have been described as singing, buzzing, squeaking or pinging (Kolbe 1973) and have excited an interest in the possibility of tsetse sound communication. Indeed, there is some evidence that tsetse flies respond to conspecific songs with a change in behaviour. Popham et al. (1976) reported that flies become more active when hearing the postfeeding song of a conspecific fly and suggested that the postfeeding song might serve as a beacon that attracts conspecifics to suitable biting sites. Increased activity was observed only in response to conspecific songs, while heterospecific songs did not have this effect (Popham et al. 1976). This might suggest a role for tsetse song in facilitating mating by bringing males and females in spatial proximity. Supporting this hypothesis, several studies demonstrated that songs differ in their frequency composition between males and females (Erickson and Moller 1975a; Popham et al. 1976). Tsetse songs have also been found to contain ultrasound components (Erickson and Moller 1975b; Saini 1983). In flight, calyptrate flies have species‐specific wing beat frequencies (Pinto et al. 2022), and it is conceivable that the high frequency of the tsetse wing beat can help identify conspecific females during the male chase. Mating songs have been described in detail in several other dipteran taxa, including drosophilids, mosquitoes and sandflies. In the two latter cases, the male song attracts females to the mating swarm, and the absence of song significantly reduces the insemination rate of *Lu. longipalpis* sandflies (Vigoder et al. 2020). Sound‐baited traps have been successfully deployed in mosquito control (Steele and McDermott 2022), yet auditory behaviours in tsetse remain under‐explored.

**FIGURE 4:**
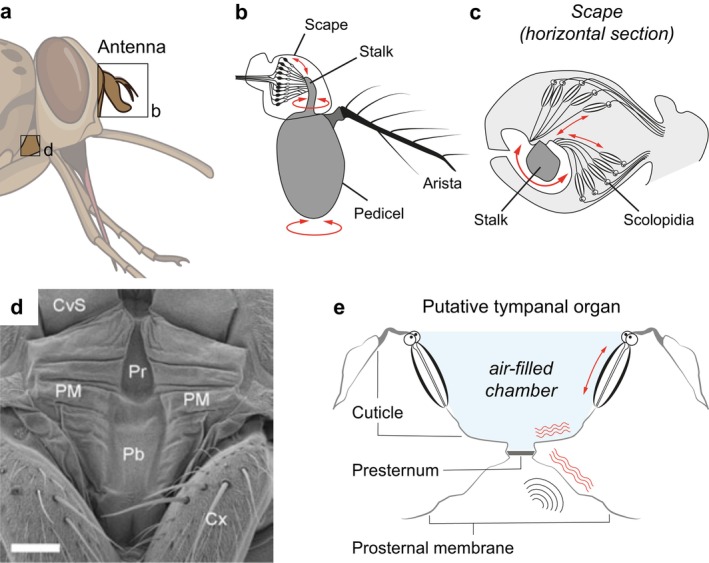
The auditory system. (a) Putative auditory organs have been described anatomically in the antenna and the prothorax. (b) The antennal auditory organ is Johnston's Organ (JO), located in the second antennal segment, the scape. Sound waves deflect the arista, which rotates the pedicel. The rotation is mechanically transmitted via the stalk to chordotonal organs in the JO. (c) Scolopidia within the JO is coupled to the stalk via an apical cap. When the cap is pulled by the moving stalk, dendrites of mechanosensory neurons within the scolopia are compressed, activating the neurons. (d) Anatomy of the prothorax. CvS = cervical sclerites, Cx = coxa of the foreleg, Pb = probasisternum, PM = prosternal membrane, Pr = presternum. Reproduced from (Tuck et al. 2009). (e) The putative tympanal organ in the prothorax. Sound waves cause the prosternal membrane to vibrate, which is transmitted to two chordotonal organs via an L‐shaped piece of cuticle. Redrawn from (Tuck et al. 2009).

Despite the potential importance of auditory signals for tsetse ecology, an auditory organ has not been unambiguously identified, and two candidates remain: an antennal sound receiver and a tympanal organ on the prothorax (Figure 4a). In fruit flies and mosquitoes, the antennal ear is located in the second antennal segment. This segment (the pedicel) contains a chordotonal organ, termed Johnston's Organ (JO). It is composed of an array of mechanosensory scolopidia whose distal segments are mechanically coupled to the base of the third antennal segment. When the third segment is deflected by sound, this deflection is sensed by the scolopidia, which transduce incoming vibrations into neural signals (Figure 4b–c) (reviewed in (Yack 2004)). This type of antennal auditory organ is specialised for detecting low‐frequency near‐field sounds, such as courtship songs in *D. melanogaster* (Bennet‐Clark and Ewing 1970) or conspecific wing beat frequencies in *Toxorhynchites brevipalpis* mosquitoes (Göpfert and Robert 2000). The *Glossina* JO has been described anatomically (Jobling 1933), and there is behavioural evidence that the tsetse antenna contains an auditory organ, as ablation of the third antennal segment abolished behavioural responses to conspecific songs (Popham et al. 1976). However, there are no published functional data on the tsetse JO.

A second putative auditory organ has been described more recently. Located on the prothorax, this tympanal organ consists of a membrane, an air‐filled chamber and two chordotonal organs that are mechanically coupled to the posterior wall of the chamber (Figure 4d–e, (Tuck et al. 2009)). Tympanal organs differ in their detection range from antennal auditory organs in that they can detect sound over longer distances and up to much higher frequencies (Yack 2004). The tsetse prothoracic membrane was tested for its responses to sound and found to be most sensitive to a frequency range of 5.3–7.2 kHz (Tuck et al. 2009)—frequencies contained in the postfeeding song of *G. morsitans* (Saini 1983). Interestingly, similar tympanal organs have been described in a group of tabanid parasitoid flies, the Ormiini, where they are used to detect sounds associated with their cricket hosts (Robert et al. 1992).

Communication is likely not the only function of tsetse sound emissions. Buzzing has been shown to be an endothermic mechanism, which warms up the fly for efficient take‐off after feeding (Howe and Lehane 1986). It is therefore possible that communication emerged as a by‐product of a primary endothermic mechanism. Similarly, wing vibrations by the female during mating have been shown to elicit behavioural changes in the male (Briceño and Eberhard 2017), but whether this is mediated by audition or mechanosensation remains to be clarified. Conceivably, tsetse flies may also respond to environmental sounds, such as sounds emitted by hosts or predators. Understanding the tsetse auditory system and the behaviours it mediates may allow us to find frequencies which disrupt crucial behaviours, such as mating or feeding. Identifying disruptive frequencies can in turn lead to new advances in tsetse repellence.

The senses and signals discussed above mediate behaviours across medium to long distances. However, after locating and alighting on a host, the decision to probe the host's skin is largely mediated by contact cues, such as temperature, taste and mechanical stimuli. Similarly, initiating copulation depends on a contact pheromone. The sensory systems that process these short‐range stimuli will be discussed next.

## 5 Senses for Short‐Range Cues: Mechanosensation

Mechanical stimuli are omnipresent and highly relevant in the world of a tsetse fly. External mechanical stimuli include air flow during flight, the subtle wing vibrations of a female during mating, and the skin ripples of a host. In addition, the fly itself generates mechanical stimuli by moving its legs or changing its posture. To sense these important cues, all flies, and indeed all insects, are equipped with mechanosensors that are widely distributed across their bodies (reviewed in (Tuthill and Wilson 2016)). However, our knowledge of how these sensors function in tsetse flies and in which situations they play a role is limited.

Different types of mechanosensors primarily sense either external or self‐generated stimuli. An important sensor for externally generated cues is the mechanosensory bristle, a hair‐like structure classified as a trichoid sensillum. Bristles are innervated by a single bipolar neuron that expresses mechanically activated ion channels in its dendrites. As the bristle is deflected by external forces, for example, when the insect touches an obstacle, the resulting mechanical strain on the dendrites opens the ion channels and causes the neuron to fire. Bristles can be rapidly or slowly adapting and are directionally selective (reviewed in Tuthill and Wilson 2016). In tsetse flies, bristles have been described on the antennae (den Otter and van der Goes van Naters 1992), wings (Baldet et al. 1992) and mouth parts (Rice et al. 1973a, 1973b).

In contrast to mechanosensory bristles, campaniform sensilla are major sensors for self‐generated mechanical stimuli, such as the deformation of the cuticle during walking or flight (reviewed in Tuthill and Wilson 2016). These sensilla are flat, dome‐shaped structures embedded into the cuticle. They are innervated by the dendrites of a single bipolar neuron. During movement, the cuticle surrounding the sensilla deforms and stretches, which flattens the dome and in turn activates the sensory neuron. Like bristles, campaniform sensilla can be rapidly or slowly adapting and can be direction‐selective if elongated into an oval shape. In tsetse flies, campaniform sensilla have been found on the wings (Baldet et al. 1992), legs (Waladde et al. 1984) and mouth parts (Rice et al. 1973a, 1973b).

**FIGURE 5:**
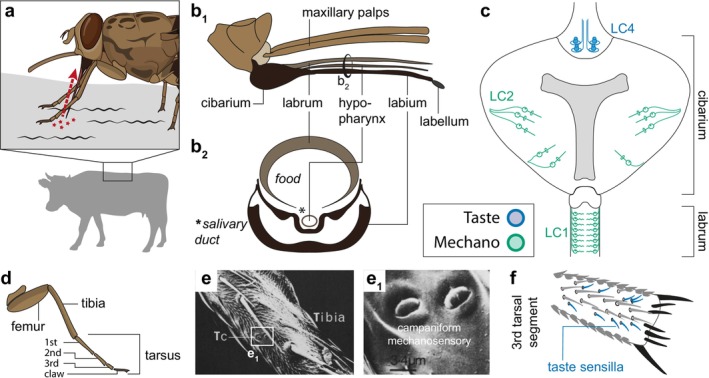
Mechanosensory and gustatory systems. (a) Mechanosensors perceive skin vibrations of hosts and deflections of the proboscis, as well as self‐generated motion. They also have a role in regulating blood flow during feeding. Gustatory sensory neurons determine the identity of the host after landing and taste the quality of the blood meal. (b) The mouthparts of tsetse consist of the cibarium, labrum (the main food channel), hypopharynx (the salivary duct), labium and labellum, along with the maxillary palps. (c) LC1 and LC2 sensilla in the cibarium and labrum are mechanosensors that sense the flow of blood through the cibarium. LC4 sensilla are taste receptors that serve as the final quality control of the blood meal before it is swallowed. Redrawn from (Rice et al. 1973b). (d) The tsetse leg below the coxa consists of the femur, tibia and the tarsus. (e) Campaniform sensilla on the tibia sense deformation of the cuticle. Reproduced from (Waladde et al. 1984) (f) Upon landing on a potential host, gustatory sensilla on the third tarsal segment perceive characteristic compounds secreted by the host. Redrawn from (Lewis 1954).

Since discussing mechanosensation in a broader context is beyond the scope of this review, we will focus on the mechanosensory systems that perceive two important stimuli (Figure 5a–c): (1) substrate vibrations, such as skin ripples of a host or a female's wing vibrations during mating, and (2) blood flow through the proboscis during feeding. In the context of sensing substrate vibrations, the campaniform sensilla on the legs have been suggested to play a role. Located on the tibia, these receptors respond to mechanical stress (Figure 5d–e, (Waladde et al. 1984)). In other insects, campaniform sensilla are proprioceptors that respond to changes in leg posture, aiding in gait and stance stabilisation (Dickerson et al. 2021). While their exact function has not been described in tsetse, it is possible that they can perceive positional changes of the leg that result from skin vibrations of a host, and that this information in combination with efferent copies is used both to sense external substrate vibrations and to help stabilise the fly's posture. Similarly, when females vibrate their wings during copulation (Briceño and Eberhard 2017), the male may perceive this signal via his tibial mechanosensors or alternatively with his JO. It has also been suggested that surface vibrations may be transmitted via the legs to the tympanic organ on the prothorax (Tuck et al. 2009). Whether this organ is primarily auditory or mechanosensory remains to be elucidated.

The mouth parts contain different types of mechanosensors, including both mechanosensory bristles and campaniform sensilla. They can be further subdivided into two groups: external and internal mechanosensors. External sensilla are located on the labium and respond to mechanical deflection of the proboscis (Figure 5a–c; (Rice et al. 1973a)). They have been proposed to sense the angle of the proboscis, as well as small relative movements between the host and the fly when the proboscis is inserted into the host. Internal sensilla are located in the labrum and cibarium (Figure 5b–c). This group of sensilla was suggested to act as flow metres, sensing the flow of the blood meal and the movement of saliva, as well as coordinating cibarial pumping (Rice et al. 1973b). Interestingly, when a fly becomes infected with trypanosomes, the parasites attach to mechanosensors in the labrum (Jenni et al. 1980), with two direct consequences: First, the parasites impair proper functioning of the mechanosensor. Second, accumulation of parasites partially blocks the labrum and thus restricts the amount of blood that can be ingested, decreasing the flow rate. Taken together, both result in reduced (real and perceived) blood flow, leading the fly to feed more often and for longer to compensate for incomplete blood meals. By attaching to mechanosensors, trypanosomes therefore influence their vector's feeding behaviour and induce it to feed more frequently—an adaptation likely evolved by trypanosomes, which contributes to increasing their spread (Jenni et al. 1980).

## 6 Senses for Short‐Range Cues: Thermosensation

It is now well established that mosquitoes use temperature cues to find optimal biting sites on the host's skin (Coutinho‐Abreu et al. 2022). The same is true for tsetse flies, as heat elicits biting behaviour in several tsetse species (Reinouts van Haga and Mitchell 1975; van der Goes van Naters et al. 1998). Early studies identified the antennae as the primary sites housing thermoreceptors (Dethier 1957), and electrophysiological responses to temperature changes were found in the third antennal segment (den Otter and van der Goes van Naters 1992). However, the identity and exact location of the receptors are still unclear. In *Drosophila*, temperature is sensed by three types of neurons: warm‐activated cells and cells that respond to heating and cooling, respectively. A small set of warm‐activated cells is located inside the brain. These so‐called AC cells express the heat‐activated channel TrpA1 and mediate thermoregulation by setting the fly's preference for an innocuous ambient temperature (Hamada et al. 2008). The function of TrpA1 seems conserved between *Drosophila* and mosquitoes, where it mediates avoidance of high temperature (Corfas and Vosshall 2015).

Rapid responses to temperature changes are mediated by heating and cooling cells, which are located in the arista on the third antennal segment. Heating cells respond to increases in temperature, based on the temperature‐dependent activity of gustatory receptor paralogue Gr28b (Ni et al. 2013), and play a role in avoiding noxious heat. Cooling cells are activated by cooling and inhibited by heating, thus underlying both warm and cool avoidance. Their sensitivity is mediated by Brivido family TRP channels (Gallio et al. 2011) as well as the ionotropic receptors IR21a, IR25a and IR93a (Budelli et al. 2019). Interestingly, IR21a mediates heat seeking in mosquitoes, where it is expressed in coeloconic sensilla on the antenna (Greppi et al. 2020). Therefore, this receptor changed both expression site and function between *Drosophila* and mosquitoes. IR21a was shown to be expressed at low levels in the antennae of tsetse flies (Kabaka et al. 2020). Together with the electrophysiological findings of thermosensory cells in the antenna (den Otter and van der Goes van Naters 1992), this finding suggests that this receptor may also be mediating heat responses in the tsetse antenna.

In tsetse, a second set of thermosensors was identified in sensilla on the tarsi of the prothoracic leg pair (Reinouts van Haga and Mitchell 1975). These receptors were found to be phasic–tonic cooling receptors, responding with a phasic increase in spike frequency to cooling steps and a steady decrease in firing rate to slow temperature ramps. Ablation or damage to the tarsi reduced the biting response elicited in flies upon contact with heated surfaces (Reinouts van Haga and Mitchell 1975). However, this effect may be based on mechanosensation rather than thermosensation alone, and the relative influence of each component remains to be elucidated.

## 7 Senses for Short‐Range Cues: Gustation

In the studies mentioned above, it was also shown that probing increases when adding an appetitive taste stimulus to the heated surface (van der Goes van Naters et al. 1998). Chemicals on the host's skin, as well as the quality of the blood meal, are sensed by gustatory receptors. These receptors influence the last two decisions of the blood seeking behavioural sequence: whether to pierce the skin with the proboscis and whether to take a full blood meal. In laboratory experiments, *Glossina morsitans* flies landed on feeding membranes smeared with either waterbuck or ox serum with equal probability (Gikonyo et al. 2000). However, more than a third of the flies that landed on the waterbuck membrane departed before probing, a behaviour never seen on the ox‐perfumed membrane. Furthermore, those that remained on the waterbuck‐perfumed membrane changed probing sites more often, probed for longer and fed less (Gikonyo et al. 2000). This highlights that tsetse flies have mechanisms to distinguish between different hosts even at the shortest distance after they have landed on an animal.

Across insects, taste sensory neurons are located in taste sensilla belonging to three classes: taste bristles, pegs and pharyngeal sensilla. Each sensillum has a pore at the tip that enables the access of tastants. Taste sensilla are innervated by 1 to 4 taste sensory neurons, and most also have a mechanosensory neuron at the base. Taste sensilla are distributed across the body, including the mouth parts, legs and wings (Figure 1).

Sensory neuron sensitivity is conferred by different families of taste receptors: The most common and abundant are GRs and IRs, but transient receptor potential (Trp) and Pickpocket (ppk) gene families have also been described to transduce taste stimuli (Chen and Dahanukar 2020). These stimuli include classical taste modalities such as sweet, bitter, salty and sour, but also water detection and pheromone sensitivity, and mediate a wide variety of behaviours essential for the animal's survival and reproduction, including feeding, mating and egg laying (Cameron et al. 2010; Matthews et al. 2019; Dweck and Carlson 2023; Baik et al. 2024). A feature of taste neurons is that they often express multiple receptors simultaneously, providing them with broad stimulus sensitivity. In tsetse, the GR and IR families have been characterised (International Glossina Genome Initiative 2014; Macharia et al. 2016). There is a remarkable reduction in the number of gustatory GR receptors with 14 reported GRs, compared with 66 GRs in *D. melanogaster* . Most notably, tsetse has lost most—if not all—sweet sensors, consistent with their restricted diet on vertebrate hosts (International Glossina Genome Initiative 2014; Macharia et al. 2016).

As in other *Diptera*, taste sensilla can be found in the legs, mouth parts and wings of tsetse. When the legs of tsetse flies come in contact with appetitive compounds, such as components of human sweat, tsetse is more likely to begin probing the substrate with their proboscis (van der Goes van Naters et al. 1998). In the riverine species *G. f. fuscipes*, chemosensory sensilla located on the tarsi of the prothoracic legs were shown to respond to some components of human sweat, such as uric acid and a range of amino acids (Figure 5f; (van der Goes van Naters and Rinkes 1993; van der Goes van Naters and den Otter 1998)). Tarsal chemoreceptors also fulfil a role in mating: the female cuticle contains non‐volatile pheromones that are sufficient to induce mating when sensed by the male (Carlson et al. 1978, 1984, 2005; Huyton et al. 1980; Langley et al. 1987). This may explain the obvious sexual dimorphism in the number of chemosensory sensilla on the legs, which are more numerous in males than in females (D'Amico et al. 1992).

Gustatory receptors on the mouth parts of tsetse (Figure 5b–c) are exposed to the host's skin during probing, and to its tissue and blood once the skin is pierced (Rice et al. 1973a, 1973b). As the proboscis navigates the host's tissue in search of capillaries, it only stops when a capillary is severed and blood contacts the proboscis (Gordon et al. 1956). In laboratory experiments, tsetse rarely fed on saline unless it contained adenosine triphosphate (ATP), an important component of blood (Galun and Margalit 1969). Furthermore, electrophysiology experiments showed that at least some of the proboscis taste receptors respond to ATP (Rice et al. 1973b). Rice and colleagues counted 24 gustatory chemoreceptory sensilla situated internally and externally on the labella, with an additional four in the posterior cibarium (Rice et al. 1973a, 1973b)—significantly fewer than can be found on the mouthparts of *D. melanogaster* , which has approximately 70 sensilla (reviewed in (Vosshall and Stocker 2007)), but more than the 15 taste sensilla present on the mosquito labella (Baik et al. 2024). It is interesting to note that the four LC4 receptors located in the posterior cibarium are the only gustatory receptors found within the food duct and therefore suited to providing a final quality control of the blood meal before it is ingested (Rice et al. 1973b). Although the response profiles of tsetse gustatory neurons remain unknown, a recent transcriptomics study of the proboscis of male *G. morsitans* found expression of four IRs (Awuoche et al. 2017). Interestingly, IRs were also found to play a role in blood feeding in mosquitoes, where taste receptors sensitive to blood carbonation express receptor Ir7a, while integrator neurons that integrate responses to other blood components express Ir7f (Jové et al. 2020). Taken together, these findings suggest that taste neurons in the proboscis provide a final quality control mechanism for the blood meal in both mosquitoes and tsetse flies.

Finally, taste sensilla have also been described on the wings of tsetse flies and identified as taste sensors due to the presence of a terminal pore (Geoffroy et al. 1992). The number of wing sensilla does not differ between males and females but varies substantially across different species. In *D. melanogaster* , similar wing chemosensors express Ir52a, a receptor that was shown to promote courtship (He et al. 2019). Other taste sensilla on the wing respond to bitter and sweet compounds (Raad et al. 2016). While there is currently no functional data available for the wing taste organs of tsetse flies, a role in mating and/or host‐finding behaviour cannot be excluded.

## Conclusions and Outlook

Tsetse flies have been studied for over a century, in particular with respect to vector control. Re‐examining this research through the lens of sensory ecology allows us to identify future avenues for vector control through a better understanding of how tsetse flies perceive, process and integrate sensory cues (Santer et al. 2019). However, tsetse fly research can offer insights beyond vector control, for example, on understanding the convergent evolution of blood‐feeding lifestyles.

Blood feeding is a specialised and robust behaviour that has evolved independently more than 20 times in arthropods and at least 12 times within the *Diptera* (Narasimhan et al. 2017; Wiegmann et al. 2011), including the lineages of mosquitoes, horse flies, blow flies and tsetse flies (Wiegmann et al. 2011). Despite their independent evolution, all groups follow a similar behavioural sequence during host‐seeking, but whether the same sensory pathways have been co‐opted for this task remains unknown. Comparing across clades will enable us to decipher the evolutionary principles underlying adaptations to a blood‐feeding lifestyle, for example, by revealing whether the differences and similarities are based on convergent or parallel evolution. Similarly, the publication of the genomes of six tsetse species (Attardo et al. 2019) gives us the opportunity to compare across species that are closely related and share a blood‐feeding lifestyle, but feed on different hosts and have adapted to different environments. How these potentially crucial differences impinge on the evolution of sensory systems is an enticing question for future research.

We know nothing about how different sensory cues are processed and integrated in the brain of any tsetse species during host‐seeking behaviour. However, recent findings in mosquitoes may offer some insights: By combining a tethered flight assay with live calcium imaging, it was possible to show that the responses of some visual neurons in the brain's optic lobe are modulated by olfactory stimuli. In contrast, responses in the antennal lobe, the brain's primary olfactory processing centre, remained unchanged upon visual stimulation, suggesting a hierarchy in host‐relevant multisensory integration (Vinauger et al. 2019). In tsetse, the cue hierarchy for finding hosts parallels that of mosquitoes: First, long‐range olfactory and visual cues guide the fly towards its target, and then mid‐range auditory cues attract it to a suitable feeding site. Finally, short‐range cues such as temperature, taste and mechanical stimuli induce the fly to bite. Deciphering how this cue hierarchy emerges from sensory processing in the brain will be an essential next step towards a full understanding of tsetse host‐seeking strategies.

## Author Contributions

**Andrea Adden:** conceptualization, writing – original draft, writing – review and editing. **Lucia L. Prieto‐Godino:** conceptualization, writing – original draft, writing – review and editing.

## Funding

This work was supported by the Francis Crick Institute (CC2067); H2020 European Research Council (802531); Human Frontier Science Program (RGY0052/2022); Chan Zuckerberg Initiative (CP‐2‐1‐Prieto‐Godino); European Molecular Biology Organization (ALTF 55‐2021), EMBO‐YIP; Vallee Foundation; and Allen Foundation, Allen Distinguished Investigator Award.

## Conflicts of Interest

The authors declare no conflicts of interest.

## Data Availability

This is a review article and as such it contains no newly generated data.
